# Optimal Transport-Based Heterogeneous Federated Learning for Chest X-Rays

**DOI:** 10.3390/s26165059

**Published:** 2026-08-10

**Authors:** Yi Liu, Haijiang Wang, Xiaohong Qian, Jian Wan, Lei Zhang, Jie Huang, Yexin Dou

**Affiliations:** 1 School of Computer Science and Technology, Zhejiang University of Science and Technology, Hangzhou 310023, China; 222308855025@zust.edu.cn (Y.L.); zhanglei@zust.edu.cn (L.Z.); huangjie@zust.edu.cn (J.H.); 222308855011@zust.edu.cn (Y.D.); 2 Zhejiang Key Laboratory of Biomedical Intelligent Computing Technology, Hangzhou 310023, China; qianxiaohong@zust.edu.cn; 3 School of Computer Science and Technology, Zhejiang Institute of Water Resources and Hydropower Hangzhou, Hangzhou 310018, China; wanjian@zust.edu.cn

**Keywords:** federated learning, heterogeneous, optimal transport

## Abstract

In some federated learning (FL) scenarios, discrepancies in local client devices result in inconsistent image resolutions, which motivates clients to adopt models with different depths and widths. Existing heterogeneous federated learning methods struggle to maintain model accuracy while preserving computational efficiency. To tackle this issue, this paper proposes a heterogeneous federated learning framework based on optimal transport (OT) and cross-layer alignment. The framework addresses the inconsistency of model depth via cross-layer alignment, fuses parameters of layers with different widths using optimal transport, and develops an aggregation strategy for multiple heterogeneous models. Experiments demonstrate that our method can improve model accuracy by up to 1.65% while maintaining satisfactory efficiency.

## 1. Introduction

**Figure 1:**
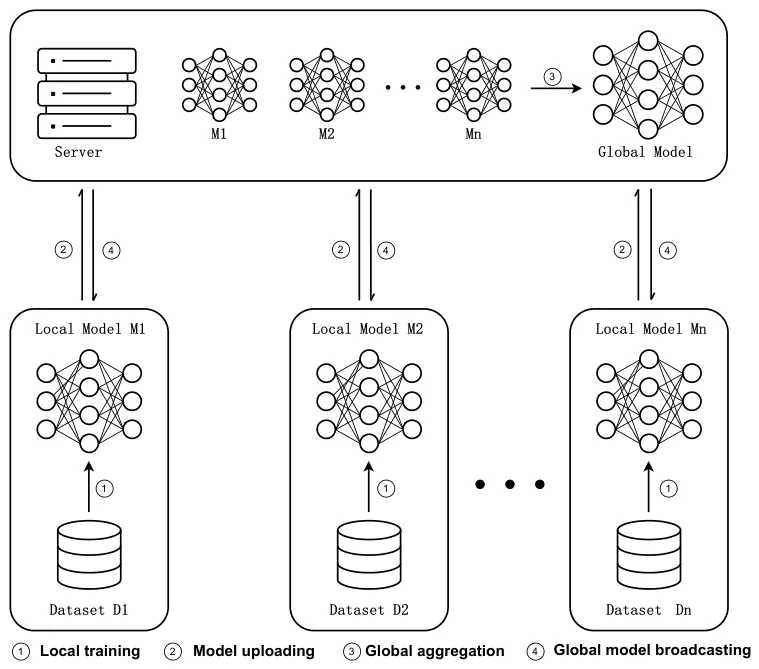
The framework of federated learning.

FL is a promising distributed collaborative learning paradigm that enables multiple participants to jointly train a global model without directly sharing raw local data, as illustrated in Figure 1.

In the first step, each client trains its local model using its own local data. Next, each client only uploads model parameters to the central server. Then, the central server combines local models uploaded by clients into a global model via parameter averaging. Finally, the central server distributes the global model to all clients as the initial model for the next communication round. The system repeats the above steps until the global model converges.

Instead of centralizing scattered private data from different clients, FL allows each participant to conduct independent local model training and only upload model parameters or intermediate gradients for global aggregation, which effectively breaks data barriers and reduces the risk of sensitive information leakage. Owing to its inherent privacy protection and distributed training properties, FL [1] has been extensively adopted in privacy-sensitive scenarios including financial risk assessment and edge computing, especially in the field of intelligent healthcare.

With the rapid development of smart healthcare, chest X-ray imaging has become a core basis for the screening and diagnosis of pulmonary diseases. Such medical data are highly private and sensitive, strictly regulated by laws [2], regulations, and medical ethics, and cannot be centrally collected or jointly trained across institutions, making distributed and privacy-preserving collaborative learning an inevitable choice. Meanwhile, medical institutions at all levels differ significantly in imaging equipment models, image resolutions, data distribution scales, and terminal computing resources [3]. As a result, institutions can only deploy local heterogeneous models with distinct network depths, widths, and architectures, which imposes a rigid requirement on FL targeting such scenarios: it must support secure and efficient aggregation under dual heterogeneity of the model depth and width.

However, existing FL methods cannot adapt to this real-world medical scenario. First, traditional FL frameworks require all clients to use an identical model structure and only support homogeneous aggregation, making them unable to handle heterogeneous models with different network depths and neuron counts. Second, mainstream heterogeneous learning approaches either rely on extra public data that are difficult to obtain in medical scenarios or incur excessive computation and prolonged runtimes, thus limiting practicality. Third, OT-based model fusion only resolves network width heterogeneity, ignores depth inconsistency caused by mismatched layer functions, and supports only pairwise fusion, which cannot be directly extended to multi-client and multi-institution federated learning scenarios.

To tackle the aforementioned problems, we propose an optimal transport-based heterogeneous federated learning (OHFL) method that allows clients to adopt models with personalized depths and widths and can simultaneously balance model accuracy and aggregation time:

- We adopt cross-layer alignment to establish functional correspondences between layers of different network depths, which resolves model depth heterogeneity in federated learning.
- We introduce OT theory to achieve parameter alignment and knowledge fusion for layers with different neuron counts, which solves model width heterogeneity in federated learning.
- We design the coupling alignment strategy with progressively increasing model depth (CID) to extend pairwise fusion to multi-model aggregation, thereby enabling efficient aggregation of multi-client heterogeneous models in FL.

## 2. Related Works

### 2.1. Heterogeneous FL

FL was first proposed by researchers at Google [4] as a novel distributed learning framework. To date, it has been extensively deployed in numerous application scenarios, such as healthcare [5], the Internet of Things (IoT) [6], edge computing [7], financial services [8], and smart city construction [9]. Nevertheless, heterogeneity has always been an unavoidable bottleneck restricting the practical deployment of FL. This work mainly concentrates on two core heterogeneous challenges in FL, namely data heterogeneity and model heterogeneity.

To address data heterogeneity, Li [10] introduced a proximal term to the local objective function, constraining client updates from deviating too far from the global model and significantly improving convergence under non-independent and identically distributed (non-IID) data. Karimireddy [11] proposed using control variates to compensate for client gradient bias, mitigating client drift caused by heterogeneous data. Reddi [12] developed adaptive optimizers to dynamically adjust client learning rates, accommodating varying data distributions. While these algorithms alleviate some challenges posed by data heterogeneity, they all assume that clients share the same model architecture.

In practice, many application scenarios require clients to retain models with different architectures. However, the aforementioned methods fail to address these issues. To tackle model heterogeneity, Yao et al. [13] split each client model into a private heterogeneous feature extractor and a globally shared classification head and employed mapping layers to align the output feature dimensions of different networks. Nevertheless, this method only supports partial model heterogeneity. Li [14] was the first to introduce knowledge distillation (KD) into FL, allowing clients to train entirely different models. They generated prediction logits on a local public dataset, which were then averaged by the server and redistributed to guide local training. On this basis, Li et al. [15] integrated logit distillation and feature KD to further boost model performance. While these approaches effectively resolve model heterogeneity, they rely on an additional public dataset, which is often impractical to obtain. Zhang [16] later proposed training a generator using an ensemble of client models to synthesize data, eliminating the need for an external dataset in KD. Similarly, Niu et al. [17] allowed clients to only upload feature statistics. After aggregating these statistics to obtain global feature statistics, clients then leverage the generative model together with the global statistics to synthesize images for local model training, thereby realizing complete model heterogeneity across all clients. However, approaches that adopt generative models to address heterogeneity in FL introduce extra time overhead for both generator training and synthetic data generation, which degrades the overall communication efficiency.

In summary, existing heterogeneous FL methods suffer from distinct limitations. They either enforce model homogeneity and cannot support network structures with different depths and widths, or they rely on public datasets with high computational costs. None of these approaches can effectively realize direct aggregation of heterogeneous models with inconsistent network depths and widths, which makes them difficult to adapt to privacy preserving collaborative training requirements in medical imaging scenarios such as chest X-rays.

### 2.2. Optimal Transport

OT theory was first proposed by the French mathematician Gaspard Monge in the mid-18th century, originally aimed at solving the mass transportation problem with minimal cost. In recent years, the introduction of the Sinkhorn algorithm [18] has greatly reduced the computational complexity of OT, making it widely concerned in the machine learning community.

Courty et al. [19] first introduced OT into machine learning domain adaptation and pioneered its use in unsupervised scenarios. They modeled two different data sources as probability distributions and aligned them via an optimal transport matrix, laying a core foundation for follow-up studies. Yan [20] realized heterogeneous domain alignment by designing conditional distribution matching and entropy regularization, introducing label information to facilitate cross-domain knowledge transfer with disparate feature spaces. Nevertheless, direct OT application may cause negative transfer and boundary classification errors. To tackle this issue, Xu [21] adopted spatial prototype and intra-domain structural features to dynamically quantify cross-domain sample differences. Hamri [22] further integrated target domain structural cues into the transport matrix by combining Wasserstein-spectral clustering and hierarchical optimal transport.

Sidak [23] first introduced OT into model fusion to overcome the drawbacks of traditional neuron-aligned fusion. By treating neural network layers as probability distributions and aligning them layer by layer, this method enables one-shot fusion of networks with different widths and partially alleviates model heterogeneity. On this basis, Chiang [24] applied OT-based fusion to federated learning and proposed progressively coupled one-shot FL, which aligns models from low accuracy to high accuracy within one communication round and achieves better performance than knowledge distillation-based methods. Zhou [25] extended this idea to federated class-incremental learning and realized heterogeneous knowledge integration by projecting the global model into local feature spaces. Imfeld [26] further adapted OT-based fusion to Transformer architectures and realized efficient one-shot fusion.

Although OT-based model fusion can handle networks with different widths, existing methods still suffer from two critical limitations. First, they only support pairwise model alignment and cannot be directly extended to multi-client federated scenarios. Second, these methods ignore layer function mismatching caused by inconsistent network depths and thus cannot simultaneously solve the problem of aggregating heterogeneous models with different depths and widths in FL.

## 3. Methods

### 3.1. Layer Alignment

**Figure 2:**
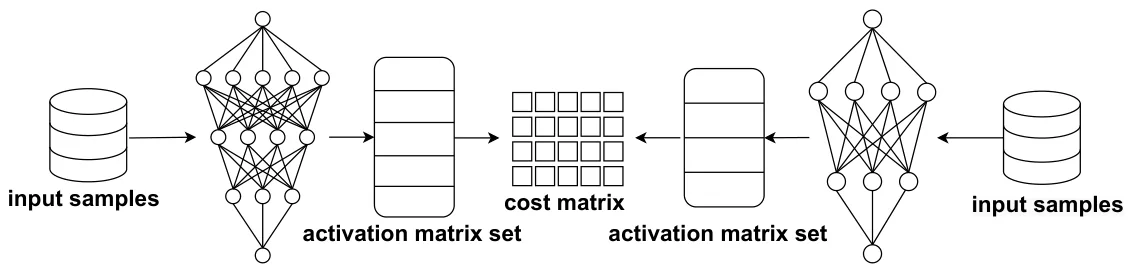
The process of finding the cost matrix.

Layer alignment is a problem of great significance in heterogeneous model fusion. For two models with identical structures, the layers at the same positions exhibit corresponding functionalities; however, for models with different structures, layers at the same positions may not share equivalent functional roles. Identifying the functional correspondence between layers of different networks is therefore critical to achieving effective fusion of the two networks. The core essence of layer alignment lies in establishing a functional mapping that matches each layer of one network to exactly one layer of the other network with different depths. To achieve this goal, we first needed to obtain effective layer representations for both networks. As shown in Figure 2, we adopted the pre-activation matrix of each layer to represent the corresponding network layer. Specifically, the pre-activation matrix was generated by feeding both networks a set of identical input samples and concatenating the outputs of each layer across all samples. By calculating the distance based on Linear CKA [27] between the pre-activation matrices of corresponding layers from the two networks, we could quantify the similarity between these layers, which was then used to construct a cost matrix *C*. Specifically, for two centered pre-activation matrices $\hat{X}$ and $\hat{Y}$ generated from the same input samples, the Linear CKA similarity metric is formulated as follows:

(1) $$C_{ij}=1-\frac{{∥{\hat{Y}}^{⊤}\hat{X}∥}_{F}^{2}}{{∥{\hat{X}}^{⊤}\hat{X}∥}_{F}\cdot ∥{\hat{Y}}^{⊤}\hat{Y}∥},$$

where ${∥\cdot ∥}_{F}$ represents the Frobenius inner product. For two models A and B, each element $C_{ij}$ in the cost matrix *C* represents the cost associated with aligning the i-th hidden layer of model A with the j-th hidden layer of model B. It is worth noting that the first and last hidden layers of the two models are aligned by default. Subsequently, we utilized the cost matrix *C* to determine the alignment relationship *a* between the hidden layers of the two networks, where a(i)=j indicates that the i-th hidden layer of model A corresponds functionally to the j-th hidden layer of model B. To obtain the optimal alignment relationship, the following conditions must be satisfied: a(1)=1, a(μ)=ν, and $S_{\mu \nu }$ are minimized. Here, μ and ν denote the number of hidden layers in the two respective networks, and $S_{\mu \nu }=\sum_{i=1}^{\nu }C_{i,\alpha (i)}$ represents the total cost required to align all hidden layers. The optimal alignment relationship *a* was thus obtained by minimizing $S_{\mu \nu }$.

### 3.2. Layer Coordination

**Figure 3:**
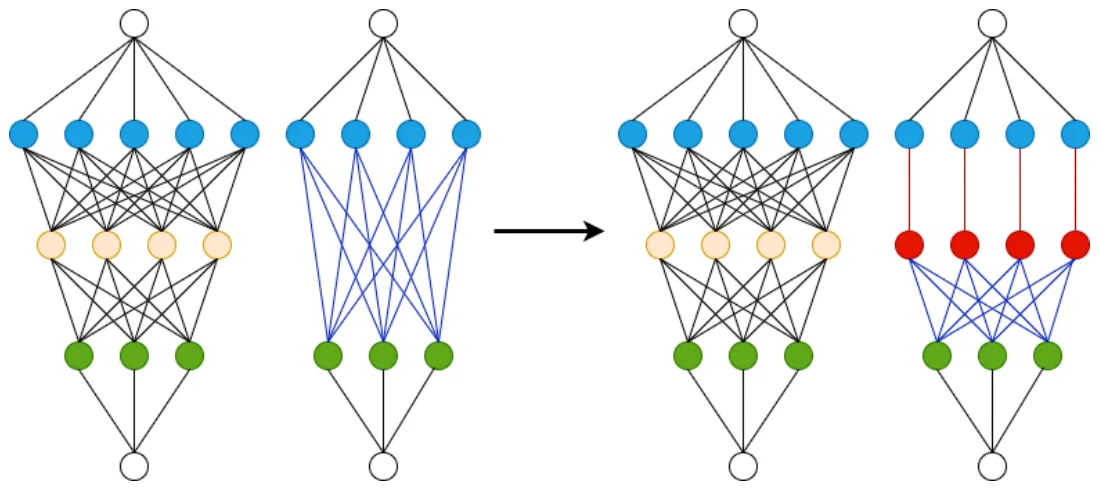
The process of adding layers.

After obtaining the layer alignment relationship between the two models, we needed to balance the number of layers in both models to facilitate subsequent fusion operations. Here, we achieved this by adding new layers to the model with fewer layers. Figure 3 illustrates the specific operation. For the two networks, layers with neurons of the same color represent functionally similar layers that have been aligned.

The operation of balancing the number of layers involves adding a new layer to the network with fewer layers. The number of neurons in the new layer is the same as that in its preceding layer, and the weight matrix of the new layer is an identity matrix, as shown in the red part of the figure. The weight matrix between the new layer and the next layer is represented by the original weight matrix, as indicated by the blue lines. The advantage of this method is that it does not alter the original parameters of the model being operated on, and adding an identity layer in this way does not affect the model’s output. The process described in Figure 3 only represents the case where the two models differ by one layer. If the difference in the number of layers is more than one, then the above process is repeated to achieve balance. However, it should be noted that this method of adding layers is currently only applicable to layers of the same type, such as layers that are both convolutional layers or fully connected layers.

If a shallower network better suits one’s needs, or if one needs to align a deep network with a shallower one, then we must perform a merging operation on the deep network. The core idea behind merging is the same as that of adding layers to ensure that the input and output of the modified network layers remain unchanged. Based on this principle, the fundamental operation for merging involves multiplying the weight matrices of the two layers to be merged. It is important to note that this process must also account for any activation layers involved, requiring an estimation of the corresponding matrix based on the specific activation function used. However, since the merging procedure is relatively complex and not applicable to our specific scenario and algorithm, we will not elaborate on it in detail here.

### 3.3. Mathematical Formulation of OT

OT gives a way to compare two probability distributions defined over a ground space S, provided an underlying distance or, more generally, the cost of transporting one point to another in the ground space. We will next focus on introducing the discrete form of optimal transport theory.

For two discrete probability measures a and b, we have the following definition:

(2) $$a=\sum_{i=1}^{n}\alpha_{i}\delta \left(x^{\left(i\right)}\right),\;b=\sum_{j=1}^{m}\beta_{j}\delta \left(y^{\left(j\right)}\right)$$

where δ(·) represents the Dirichlet function and $\alpha_{i},\beta_{i}$ denote the probabilities corresponding to each point. They satisfy the following respective conditions:

(3) $$\sum_{i=1}^{n}\alpha_{i}=1,\;\sum_{j=1}^{m}\beta_{j}=1$$

Let $C_{ij}$ represent the cost of moving a unit mass from point $x_{i}$ to point $y_{j}$. Based on the above definition, the optimal transport problem between probability measures a and b can be expressed as follows:

(4) $$OT\left(a,b,C\right)=\min_{T}∥TC∥$$

where ∥·∥ represents the Frobenius inner product, *T* represents the transport matrix, and $T_{ij}$ denotes the mass transported from point $x_{i}$ to point $y_{j}$. In this work, we utilize the Sinkhorn algorithm mentioned in Section 2.2 to efficiently compute an approximate transport matrix *T*.

### 3.4. Balance Two Neural Network Layers with OT

The discrete OT problem aims to find a transport matrix *T* to align two probability measures. Sidak proposed treating the neuron distributions of corresponding layers in two networks as two probability distributions and aligning these distributions through OT to achieve a functional soft alignment between neurons. However, directly manipulating neurons is not feasible. Below are the steps to achieve functional alignment of neurons by operating on the weight matrices.

**Figure 4:**
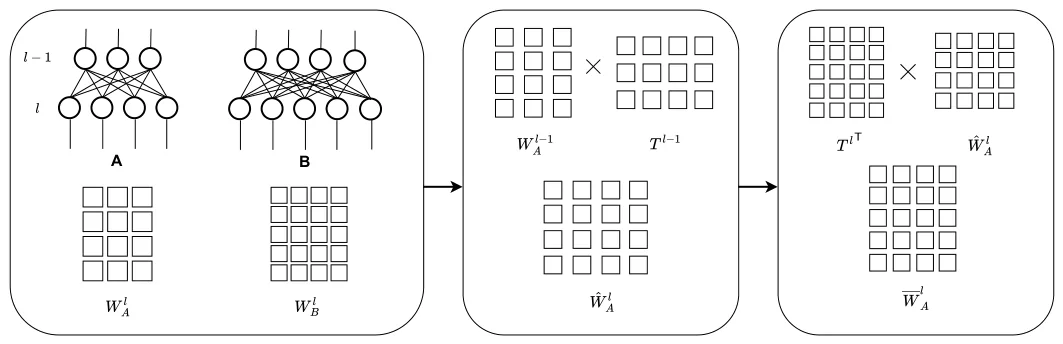
The process of OT fusion.

Suppose we want to align the *l*-th layer of model *A* and model *B*, and the previous layers have already been aligned, as we adopted a layer-wise alignment scheme where the first layer was aligned by default. The weight matrices of the current layer for the two models are $W_{A}^{l}$ and $W_{B}^{i}$. The OT matrices for the current layer and the previous layer are $T^{l-1}$ and $T^{l}$, respectively. If we assume that model *A* is to be aligned with model *B*, as shown in Figure 4, then the first step is to multiply the current layer’s transport matrix $W_{a}^{l}$ of model *A* by the optimal transport matrix $T^{l-1}$ of the previous layer to obtain ${\hat{W}}_{A}^{l}$ (${\hat{W}}_{A}^{l}=W_{A}^{l}T^{l-1}$). After this operation, $W_{A}^{l}$ and $W_{B}^{l}$ will have the same number of columns, which aim to align the number of inputs in the two layers. Next, the transpose of the current layer’s transport matrix ${T^{l}}^{T}$ is multiplied by $W_{A}^{l}$ to obtain the final aligned weight matrix ${\bar{W}}_{A}^{l}$(${\bar{W}}_{A}^{l}=\;({T^{l}}^{T}{\hat{W}}_{A}^{l}$). This matrix has the same shape as $W_{B}^{l}$, allowing their parameters to be fused using a weighted average.

### 3.5. Multi-Model Aggregation Strategy

By aligning and generating layers to balance two models of different depths and then using OT theory to fuse the weight parameters of the two balanced models with different widths, the fusion of two heterogeneous models is achieved. However, in the context of federated learning, the number of clients is typically three or more. To efficiently and accurately fuse multiple models, we developed a multi-model aggregation strategy that achieves knowledge fusion across multiple models by progressively aligning shallow models with deeper ones name coupling alignment strategy with progressively increasing model depth.

Specifically, local clients upload their trained models to the server. The server first aggregates models of the same depth into a new model, which effectively reduces redundant operations when performing layer balancing between models of identical structures and those of different structures. After performing layer operations on all models, a list containing multiple models of varying depths is obtained, with each depth corresponding to only one model. Subsequently, the model list is sorted in ascending order of depth. The shallowest model in the list is selected as the initial model, which is then aligned and fused with the next model in the sequence using the aforementioned methods to generate a new model. This new model then becomes the initial model, and the process is repeated until all models have participated in the fusion. The final resulting model is distributed as the global model to the local clients. The clients fine-tune this model using their own data to meet their specific requirements. The pseudocode for this process is detailed in Algorithm 1.
**Algorithm 1** Multi-model aggregation strategy.**Require:** All clients k=1,…,K with local dataset $D_{k}$
  1:**1. Local Training Stage:**  2:**for** k=1 **to** *K* **do**  3:    **for** t=1 **to** *T* **do**  4:        $w_{k}^{t+1}\leftarrow \mathrm{LocalUpdate}\left(k,w_{k}^{t}\right)$  5:    **end for**  6:    Upload local model $w_{k}$ to central server  7:**end for**  8:**2. Cross-Layer Model Aggregation Stage:**  9:Fuse models with identical depths to generate one representative model per depth10:Sort fused models by depth dimension: m[1,2,…,M] (M<K)11:$W_{\mathrm{initial}}=m\left[1\right]$12:**for** i=2 **to** *M* **do**13:    Execute cross-layer alignment and dimension balance between $W_{\mathrm{initial}}$ and m[i]14:    Fuse two heterogeneous models via optimal transport $\to W_{\mathrm{intermediate}}$15:    $W_{\mathrm{initial}}\leftarrow W_{\mathrm{intermediate}}$16:**end for**17:Obtain global aggregated model: $W_{\mathrm{global}}=W_{\mathrm{initial}}$18:Broadcast $W_{\mathrm{global}}$ to all participating clients19:**3. Local Fine-tuning Stage:**20:**for** k=1 **to** *K* **do**21:    Fine-tune the received global model $W_{\mathrm{global}}$ with private local dataset $D_{k}$22:**end for**


### 3.6. Applications of OHFL in the Medical Field

As previously mentioned, FL has broad applications in numerous domains, and its utility in the medical field is particularly emphasized. Given the heightened sensitivity of healthcare data to privacy concerns, it is virtually impossible to aggregate data from various medical institutions for traditional centralized training. Consequently, FL emerges as an ideal solution to facilitate collaboration among healthcare providers without compromising data confidentiality.

However, in practice, medical institutions are often geographically dispersed. The data they collect from patients may originate from heterogeneous sources. Variations in data dimensions, features, and distributions are attributable to differences in acquisition devices across institutions. These variations often lead to suboptimal performance with conventional federated learning approaches. Furthermore, disparities in data distributions, objectives, or computational capabilities may compel different institutions to maintain structurally distinct models, which cannot be aggregated using traditional federated learning frameworks.

To address these challenges, we propose a novel federated learning framework that is well suited for medical image recognition tasks such as chest X-ray diagnosis. Our framework enables participating institutions to train structurally heterogeneous models within the same model family, thereby accommodating their unique data characteristics, hardware constraints, and task requirements. These architecturally diverse models are subsequently aggregated into a global model during the serving phase. Participating healthcare providers can directly deploy this global model or further refine it through additional training.

**Figure 5:**
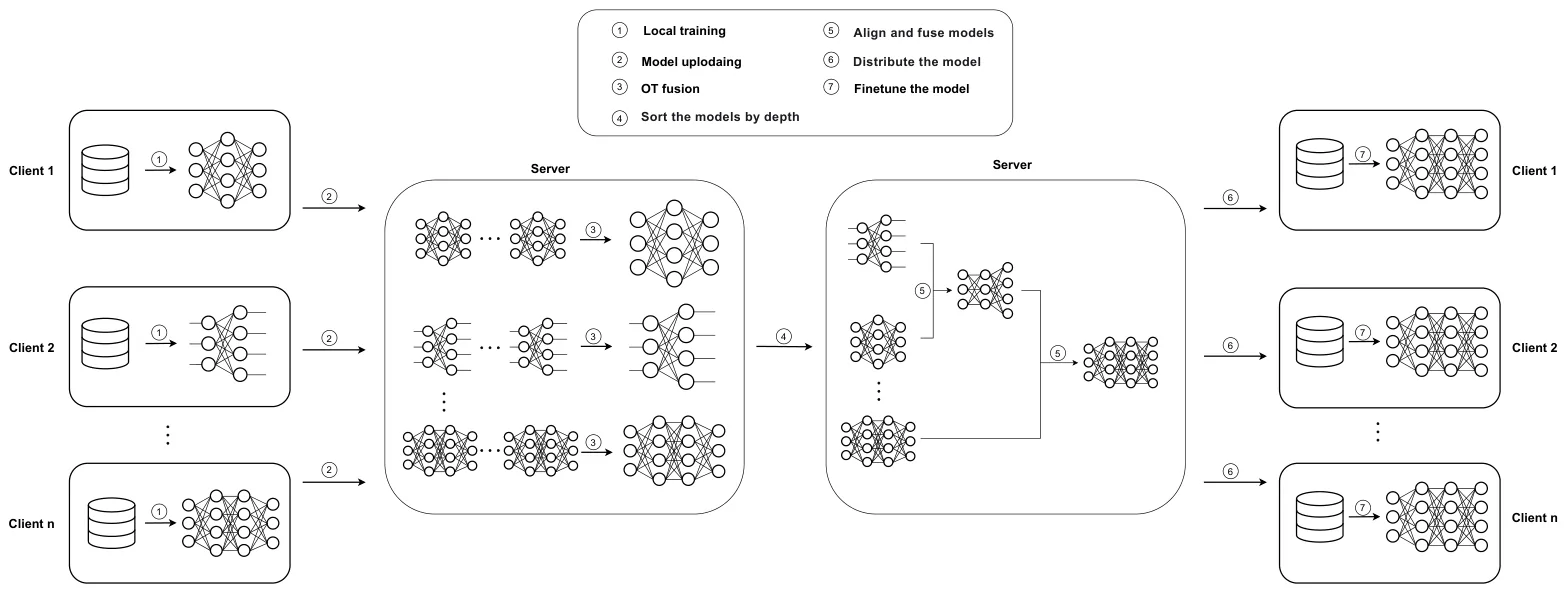
The framework of OHFL.

As illustrated in Figure 5, each healthcare institution initially trains a local model using its own medical data. During this phase, no inter-institution communication occurs; the models are trained solely based on local requirements. When participating in federated learning, the institutions upload their trained models directly to the central server. Since the models are already trained locally, this step minimizes the time overhead caused by the heterogeneity of the system. The server then aggregates the uploaded models using the methodology described earlier and redistributes the global model back to all participants. Each institution fine-tunes the global model on its local data to better adapt to the institution-specific feature distribution. The fine-tuned model can be deployed directly or used to guide downstream tasks, such as serving as a teacher network in knowledge distillation or imitation learning. Specifically, the entire process requires only one round of communication between the institutions and the server, significantly reducing the risk of data leakage while maintaining computational efficiency.

### 3.7. Complexity Analysis

Regarding notation, m denotes the number of hidden layers of the deep model, n denotes the number of hidden layers of the sampled data used to calculate layer similarity, p represents the maximum number of neurons per layer, and M stands for the count of models with distinct depths. In the layer alignment phase, the time complexity of cost matrix construction is $mnt^{2}p$, and the complexity of optimal transport fusion after layer balancing is $np^{2}\log p$. The CID aggregation strategy repeats the above two-model fusion process M−1 times, and thus the overall time complexity of server-side aggregation in OHFL is O((M−1)$\left(mnt^{2}p+np^{2}\log p)\right)$. In general, the value of *t* is relatively small, while *p* dominates the complexity. The growth of *p* will drastically increase the computational complexity.

## 4. Experiment

### 4.1. Settings

All experiments were conducted on a single RTX A6000 GPU (NVIDIA Corporation, Santa Clara, CA, USA) with 48 GB of memory. In the local training phase, we used the stochastic gradient descent (SGD) optimizer with a learning rate $\eta_{S}=0.01$ and momentum of 0.9. The local training epoch count was 20, and the local batch size was 16. In the aggregation phase, we selected the geometric ensemble type to be “wts”. The Sinkhorn entropic regularization coefficient ε = 0.01, and the chosen sinkhorn type was “normal”. During fine-tuning, we set the learning rate $\eta_{ft}=0.001$ and adopted a fine-tuning epoch count of 50. All experimental results presented in the tables were averaged over five independent runs with unified random seeds.

### 4.2. Datasets

**Figure 6:**
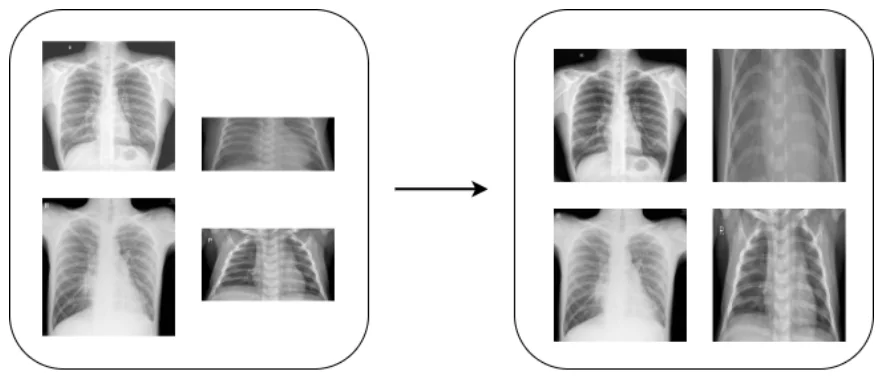
Data before and after being resized.

In the experimental section, we adopted three datasets: a chest X-ray dataset, MNIST, and CIFAR-10. The chest X-ray dataset was sourced from https://www.kaggle.com/paultimothymooney/chest-xray-pneumonia (accessed on 23 March 2025), which contains a total of 5856 chest X-ray images. The image sizes range from 384×127 to 2916×2583, and the dataset comprises two categories: 1583 normal chest X-ray images and 4273 pneumonia images. Figure 6 illustrates some significant differences in the distribution and size of the dataset before and after resizing. To simulate the discrepancy in local data sizes among different hospitals in real-world scenarios, we divided all normal and pneumonia data based on the image width into six groups sorted from smallest to largest, as shown in Table 1. After this division, the images stored on each client varied greatly in size. For each client, we split its data into training, validation, and test sets at a ratio of 80%:10%:10%. Due to the class imbalance in the samples, we adopted online data augmentation, including random horizontal flip, limited random rotation, and slight random translation, for the local normal samples during the training phase. The MNIST dataset contains binary images of handwritten digits. There are 60,000 training images and 10,000 testing images in the MNIST dataset. The CIFAR10 dataset consists of 60,000 32 × 32 color images in 10 classes, with 6000 images per class. There are 50,000 training images and 10,000 test images in the CIFAR10 dataset. For the two datasets, data were equally partitioned among six clients, with 10% of each client’s local training data reserved for validation.

### 4.3. Model and Baseline Methods

In this paper, three models are used: a convolutional neural network (CNN [28]), Visual Geometry Group 11 (VGG11 [29]) and adaptive differential privacy-based federated learning (DPFL [30]). The CNN backbone consisted of three convolutional layers and two fully connected layers (CNN: input layer → conv3-8 → conv3-16 → conv3-32 → fc-1000 → fc-500 → output layer). We adopted this model to validate the performance of our heterogeneous FL algorithm on shallow network architectures. Additionally, we constructed two homogeneous models with different depths by removing one convolutional layer and adding one fully connected layer. The VGG11 model was relatively deep with a larger number of parameters. We utilized it to verify the performance of our proposed algorithm on deep network architectures. Similarly, we constructed two homogeneous models with distinct depths by removing one convolutional layer and adding one fully connected layer. DPFL is a convolutional neural network that achieves excellent performance within the FL framework on chest X-ray datasets. We adopted this model to validate the performance of our algorithm on architectures tailored for chest X-ray images, and we constructed two homogeneous models with distinct depths by adding one convolutional layer and one fully connected layer.

For each model family containing three distinct architectures, we assigned the shallowest model to Client 1 and Client 2, the medium-depth model to Client 3 and Client 4, and the deepest model to Client 5 and Client 6. This allocation scheme matched the data size owned by each client. All local models on each client were trained from scratch without loading any pretrained weights.

In addition to the proposed method, three other methods were employed. The first was FedAvg [7], a classical homogeneous federated averaging baseline. For fair heterogeneous comparison, we enforced all participating clients to maintain the identical most complex local model architecture in this baseline set-up. The second was FedMD [14], a knowledge distillation method that requires a public dataset, and the final one was Dense [16], a federated knowledge distillation method that leverages generators and requires no public datasets. Meanwhile, we compared our CID with three alternative aggregation strategies. The coupling alignment strategy with progressively decreasing model depth (CDD) is similar to our proposed CID method, but its sorting process runs from deep models to shallow models. Accordingly, the final global model adopts the network structure of the shallowest model. The coupling alignment strategy with progressively increasing model accuracy (CIA), inspired by Chiang’s CODE approach [24], first ranks all heterogeneous models according to their accuracy and then executes coupled fusion sequentially from the lowest accuracy to the highest accuracy. Finally, the RAND strategy is a common method that extends the two-model fusion method to multi-model fusion. Specifically, it randomly selects one model from *n* heterogeneous models as the baseline model. For each remaining model, it performs pairwise fusion with the baseline model and then aggregates the resulting n−1 fused models through parameter averaging.

### 4.4. Results

To fully verify the performance of the proposed OHFL algorithm, we first conducted experiments on the chest X-ray dataset. On the one hand, we demonstrate the performance gaps between OHFL and other comparative methods across diverse network architectures. On the other hand, we compared OHFL with baseline approaches in terms of aggregation speed to reveal its advantages and disadvantages. Meanwhile, we compared OHFL with other baseline algorithms on two public datasets, MNIST and CIFAR-10, and revealed the influence of non-IID data distribution on model performance. Furthermore, we conducted comparative experiments between our proposed CID aggregation strategy and several alternative aggregation schemes in terms of client model stability and the convergence speed of the global model. Finally, we carried out ablation studies to validate the respective contributions of the layer alignment module and the proposed CID aggregation strategy to the overall framework.

Table 2 compares the global performance of OHFL with three baseline algorithms on the chest X-ray dataset when varying the number of participating clients. From the overall trend, we observe that the global classification accuracy of all algorithms rose steadily when the number of participating clients increased from 2 to 6, and the proposed OHFL consistently achieved the best global accuracy under all settings of participating clients and across the three model architectures, which fully demonstrates the advantages of our framework for handling heterogeneous client models. The performance advantages of OHFL over baseline methods varied under different client quantities and model architectures, with accuracy gains roughly ranging from 0.3% to 1.65%.

**Figure 7:**
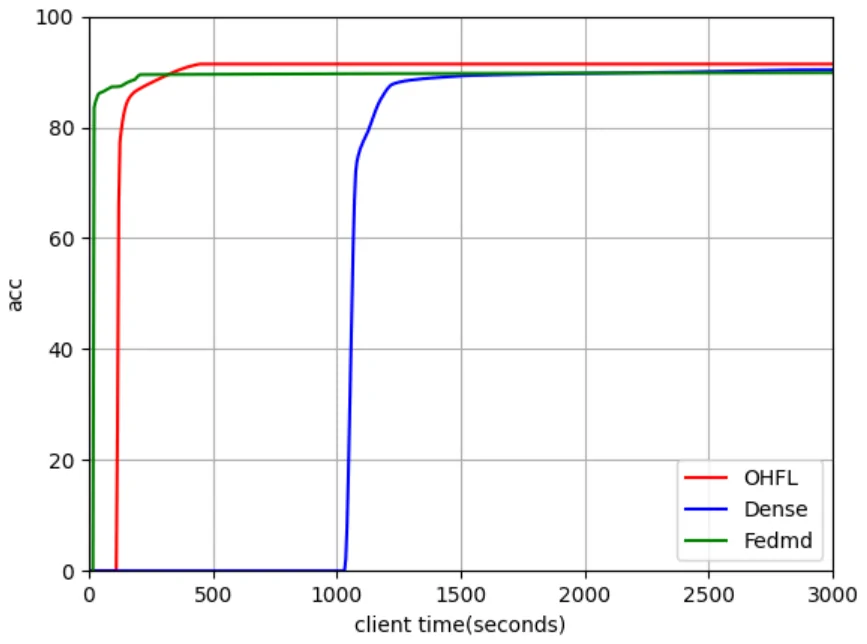
Convergence curves of different methods.

Figure 7 shows the global convergence curves of OHFL, DENSE, and FedMD, where the vertical axis corresponds to model accuracy and the horizontal axis indicates the additional time cost after local training. Compared with DENSE, FedMD and OHFL consumed much less time. Despite being marginally slower than FedMD, OHFL obtained superior accuracy, verifying that our method realizes a good balance of prediction accuracy and computational cost.

Table 3 presents the performance comparison between the proposed OHFL and baseline methods under different heterogeneity levels on the MNIST and CIFAR-10 datasets. CNN was adopted as the network model, where α denotes the Dirichlet heterogeneity level. It can be observed that OHFL achieved slight improvements over the baseline methods under mild heterogeneous settings with α=0.5. Nevertheless, under the extreme heterogeneous scenario with α=0.1, OHFL failed to adapt well to skewed data and underperformed compared with FedMD. This indicates that OHFL, designed primarily to tackle model heterogeneity, possesses limited capability to handle data heterogeneity. When the local data distributions became heavily skewed, OHFL could not outperform algorithms that are well adapted to distribution heterogeneity. This limitation motivates our future research, in which we will explore effective strategies to boost the model performance under non-IID data.

Table 4 presents the performance of the global model on each client under different aggregation strategies. We can observe that the proposed CID achieved the highest average accuracy across six clients with the smallest variance compared with other aggregation strategies. This reveals that under the CID strategy, the global model can better adapt to each individual client while maintaining relatively small performance discrepancies among clients, which satisfies the fairness principle.

**Figure 8:**
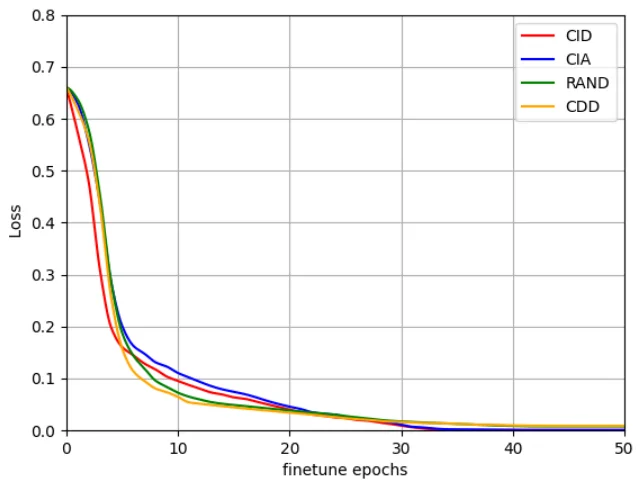
Loss convergence curves of different strategies during fine-tuning.

Figure 8 presents the loss curves of the global model during fine-tuning under different aggregation strategies. The global model under the CID strategy exhibited a faster decline in loss and achieved a lower final convergence value, which indicates that the global model aggregated by CID possessed superior initial parameters.

Table 5 shows the results of the ablation experiments. Among the three modules proposed in this work, the layer coordination module was indispensable since it served as the foundation for the subsequent aggregation procedure. Therefore, we mainly conducted experiments on the layer alignment module and the CID aggregation strategy. Here, random aggregation corresponds to the RAND aggregation strategy. Random alignment performs random matching between layers without cross-type alignment restrictions; specifically, scenarios such as aligning convolutional layers to fully connected layers are prohibited. The results demonstrate that both the layer alignment module and the CID aggregation strategy achieved considerable performance improvements for OHFL. Moreover, layer alignment exerted a greater impact on OHFL compared with CID. A plausible explanation is that layer alignment is executed in the first stage, and its effects accumulate during subsequent procedures, making it more critical.

## 5. Conclusions

This work focused on FL scenarios where clients adopt locally trained models with distinct architectures due to model size heterogeneity and varying computing resources. Existing approaches struggle to achieve a favorable trade-off between accuracy and training efficiency. Accordingly, we proposed OHFL to strike a balance between accuracy and time consumption in heterogeneous federated learning. We simulated such practical scenarios on the chest X-ray dataset to verify the effectiveness of our method, and we also discussed its limitations on the MNIST and CIFAR-10 datasets. This motivates our future research directions. On the one hand, we will explore effective strategies to strengthen the adaptability to non-IID data. On the other hand, current OT-based heterogeneous model fusion methods can hardly fuse models from different model families, which restricts the range of available model architectures for clients. We intend to develop new fusion schemes to support the fusion of models belonging to different families in future work. 

## Figures and Tables

**Table 1:** Dataset characteristics for normal and pneumonia cases.

Group	Normal		Pneumonia	
	Volume	Size (px)	Volume	Size (px)
1	233	662–1080	642	127–584
2	233	1082–1206	642	584–680
3	233	1208–1330	642	680–776
4	233	1330–1468	642	776–880
5	233	1468–1693	642	880–1056
6	238	1696–2625	643	1056–2192
Total	1583	662–2625	4273	127–2192

**Table 2:** Performance comparison across different algorithms and client numbers.

Algorithm	Model	2 Clients	4 Clients	6 Clients
OHFL	CNN	87.83±0.69	90.04±0.21	91.69±0.31
FedMD	CNN	87.35±0.95	88.84±0.33	90.09±0.22
DENSE	CNN	87.53±0.94	89.04±0.84	89.34±0.40
Fedavg	CNN	86.68±0.54	88.42±0.52	90.04±0.26
OHFL	VGG11	89.94±0.47	91.01±0.35	92.03±0.25
FedMD	VGG11	89.14±0.55	90.18±0.44	91.21±0.43
DENSE	VGG11	88.78±0.83	90.54.±0.52	90.97±0.36
Fedavg	VGG11	88.81±0.49	89.91±0.31	90.78±0.41
OHFL	DPFL	88.83±0.37	91.18±0.36	92.16±0.42
FedMD	DPFL	88.14±0.45	90.63±0.46	91.27±0.39
DENSE	DPFL	87.95±0.68	90.37±0.51	91.57±0.35
Fedavg	DPFL	88.36±0.59	90.27±0.26	91.37±0.24

**Table 3:** Performance comparison on MNIST and CIFAR-10 with different heterogeneity levels.

Algorithm	MNIST		CIFAR-10	
	α=0.1	α=0.5	α=0.1	α=0.5
OHFL	85.14±1.16	95.15±0.58	63.84±0.94	72.14±0.62
FedMD	89.36±1.34	92.49±0.47	65.29±1.13	71.78±0.59
DENSE	83.25±1.52	93.55±0.73	60.71±1.28	68.90±0.97
FedAvg	81.73±0.92	90.81±0.29	62.96±0.77	69.42±0.53

**Table 4:** Client model performance under different aggregation strategies.

Strategy	Client Number						Average	Variance
	1	2	3	4	5	6		
CID	90.79±0.27	91.14±0.33	91.52±0.27	91.65±0.39	92.05±0.40	91.74±0.46	91.48	0.203
CDD	91.24±0.17	91.10±0.25	90.81±0.38	90.93±0.31	90.13±0.62	89.94±0.57	90.69	0.236
CIA	89.35±0.57	88.93±0.51	89.62±0.74	91.11±0.79	88.78±0.69	88.59±0.75	89.36	0.753
RAND	89.98±0.49	90.02±0.56	89.54±0.62	88.76±0.71	88.07±0.79	87.90±0.68	89.05	0.939

**Table 5:** Ablation study of layer alignment and CID.

Setting	Test Accuracy
random alignment + random aggregation	84.06±1.28
random alignment + CID	84.72±0.85
layer alignment + random aggregation	89.51±0.65
layer alignment + CID	91.69±0.31

## Data Availability

The original contributions presented in this study are included in the article. Further inquiries can be directed to the corresponding author.
